# Immunoglobulin D Multiple Myeloma With Rapidly Progressing Renal Failure

**DOI:** 10.14740/jocmr2210w

**Published:** 2015-06-09

**Authors:** Jwalant Modi, Jeanne Kamal, Ahmad Eter, Suzanne El-Sayegh, Elie El-Charabaty

**Affiliations:** a Department of Internal Medicine, Staten Island University Hospital, NY, USA

**Keywords:** Immunoglobulin D, Multiple myeloma, Light chain nephropathy, Bortezomib

## Abstract

Immunoglobulin D (IgD) multiple myeloma (MM) is a very rare form of myeloma affecting less than 2% of all myeloma patients. It has a multiorgan involvement with renal failure being the key feature. We present here a case of IgD MM in a 62-year-old white male, smoker with past medical history of hypertension, who presented to emergency department with complaints of lower abdominal pain, constipation and decreased urination. Physical exam was unremarkable. Laboratory investigation showed S.Cr 5.99 mg/dL, hemoglobin 8.7 g/dL and corrected S.Ca 10.6 mg/dL. Urine dipstick showed 100 protein and TP/Cr ratio was 23. Serology was positive for serum free lambda chain level of 8,947.6 mg/L as well with free κ/λ ratio < 0.01. The results of serum and urine electrophoresis and immunofixation were also supportive of diagnosis of IgD MM. IgD level was remarkably elevated (27,300 mg/L) too. CT scan of abdomen/pelvis was negative for obstructive uropathy. Skeletal survey showed a solitary lytic lesion in the iliac crest. His kidney function deteriorated next day requiring hemodialysis. The bone marrow biopsy was positive for plasma cell hypercellularity (70-80%) and flow cytometry showed 8% monoclonal IgD lambda plasma cells. The patient was started on bortezomib and dexamethasone and he underwent bone marrow transplant 6 months later. He is doing well hematologically now but he remains dialysis-dependent. IgD MM is a very rare disease affecting younger population with poor prognosis; patients often end up on hemodialysis despite better control of the hematological component.

## Introduction

Immunoglobulin D (IgD) multiple myeloma (MM) is a rare subtype of myeloma, with an incidence of less than 2% of all myelomas [1]. It carries a poorer prognosis than other myeloma isotypes. It can cause multiorgan involvement with renal failure being the key feature, occurring in 20-40% of patients at the time of diagnosis, up to 10% requiring renal replacement therapy, and 25% later during the disease [2]. Cast nephropathy is the main underlying pathophysiologic mechanism.

## Case Report

A 62-year-old Caucasian male with history of diet controlled hypertension, dyslipidemia and smoking came to emergency department with complains of lower abdominal pain and decreased urination starting progressively over a couple of weeks. He also had constipation along with mild nausea, fatigue with diffuse joint pain and cold intolerance for almost a month.

On physical examination, his vitals were normal. He did not have any costovertebral angle tenderness. Patient was oliguric with 300 mL of urine upon Foley catheter insertion. On laboratory investigation, hemoglobin was 8.7 g/dL; his serum creatinine was 5.99 mg/dL (GFR MDRD of 10 mL/min), and albumin/globulin (AG) ratio was 0.84. He had mild hypercalcemia (corrected Ca^2+^: 10.6 mg/dL). Of note patient’s serum creatinine was 2.6 mg/dL a week ago as outpatient checked by his primary physician and his hemoglobin and creatinine were normal 1 year prior. His urinalysis showed 100 protein, positive blood and occasional mucous threads. A non-contrast CT scan of abdomen/pelvis was negative for any urinary tract calculi or hydronephrosis.

The next day, patient’s serum creatinine increased to 8.5 mg/dL. His urine total protein creatinine ratio was 23 mg/mg and ESR was 120 mm/h. He became anuric with 100 mL of urine in 24 h so the decision was made to begin renal replacement therapy with hemodialysis. Given the high clinical suspicion of dysproteinemia (bone pain, mild hypercalcemia, low AG ratio, and normocytic anemia), oncology team was consulted and the patient was started on bortezomib and dexamethasone awaiting the results of confirmatory studies.

**Figure 1:**
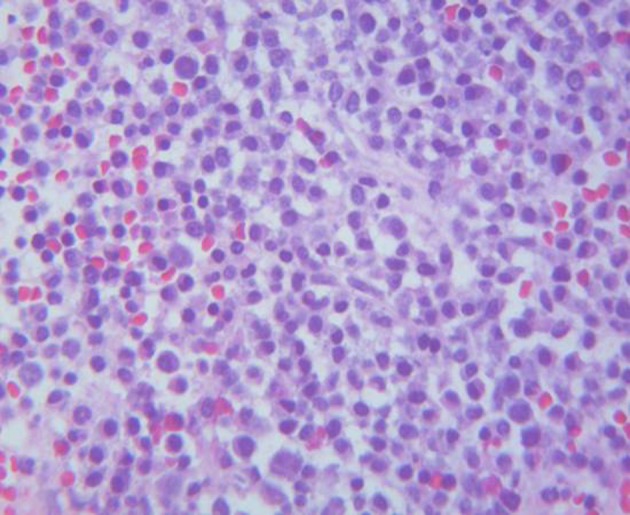
Bone marrow: plasma cells proliferation (× 400).

**Figure 2:**
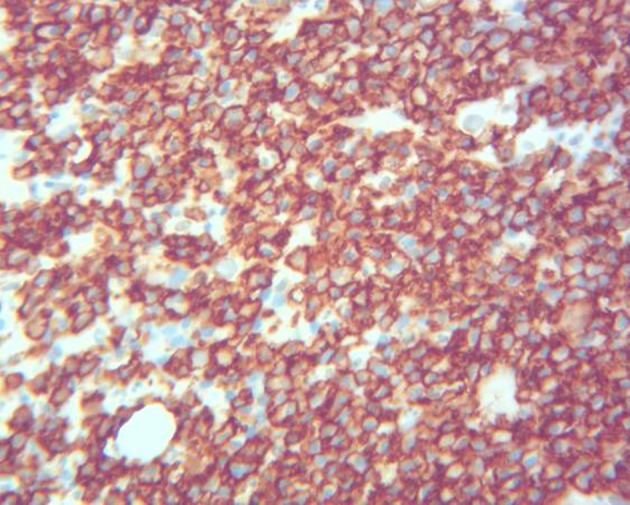
Bone marrow: CD138 immunohistochemical stain (× 400).

**Figure 3:**
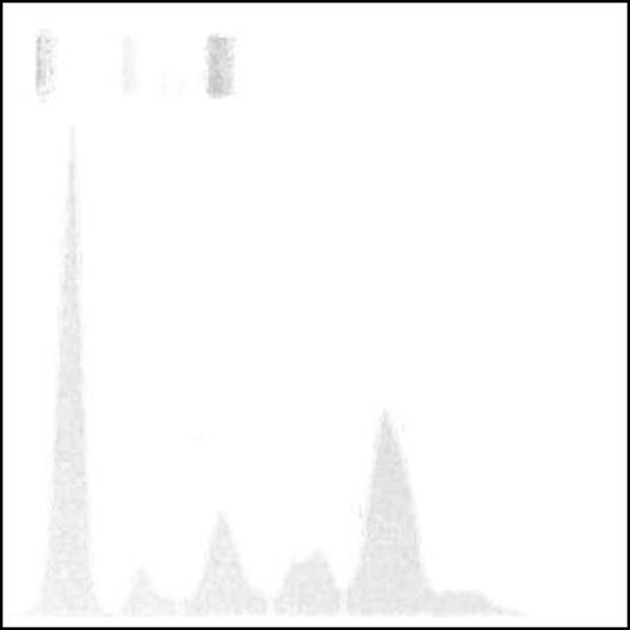
Serum protein electrophoresis: monoclonal M-spike.

Bone marrow biopsy showed hypercellularity (70-80%) with sheets and large aggregates of plasma cells (Fig. 1) with a flow cytometry positive for monoclonal IgD λ plasma cells (8% of total cells) with the following immunophenotype: CD38, CD138 (Fig. 2), cytoplasmic λ light chains and IgD. Fluorescence *in situ* hybridization (FISH) was positive for 1q21 in 7.5% of cells. Serum free λ chain level was elevated (8,947.6 mg/L) as well with free κ/λ ratio < 0.01. Serum IgD level was remarkably elevated (27,300 mg/L) too. The results of serum (Fig. 3) and urine electrophoresis and immunofixation were concordant with the diagnosis of IgD λ monoclonal light chain producing plasma cell myeloma. Patient was found to have a solitary lytic lesion measuring 0.9 cm in left iliac crest bone on the skeletal survey. Kidney biopsy was not performed in light of the confirmed clinical diagnosis of myeloma-induced light chain cast nephropathy.

The patient’s symptoms improved upon initiation of hemodialysis. He was discharged 8 days after and continued to receive hemodialysis and chemotherapy. His chemotherapy consisted of bortezomib and dexamethasone and he underwent bone marrow transplant 6 months later. Repeat serum electrophoresis, serum free κ and λ chain levels and IgD levels done 12 months after treatment were near normal. The patient has achieved hematological remission of myeloma but he remains dialysis dependent.

## Discussion

IgD MM has different characteristics than other myeloma isotypes. IgD MM is rare [1], has an onset at a younger age with poor prognosis and a median survival of less than 2 years prior to the availability of novel agents and use of autologous stem cell transplantation (ASCT) [3]. For instance, 1q21 as in our patient has adverse prognosis for event free and overall survival. Compared to IgG and IgA subtypes, serum concentration of IgD is much lower. Thus, it may only show a small or absent M-spike on SPEP, or an unidentified Ig isotype posing a diagnostic challenge. Worsening kidney function of unknown cause along with diffuse bone pain as in our patient should raise the suspicion of IgD isotype MM as most of the cases are often diagnosed late in the course of the disease and this delay in diagnosis contributes to poor survival [4, 5].

Light chain cast nephropathy is the most common pathophysiologic mechanism leading to renal failure in this disease. The filtered monoclonal light chains form intratubular casts and obstruct the tubular flow, incite foreign body reaction and cause tubular fibrosis. In addition, light chains can also cause direct toxicity to proximal tubular cells and intracellular crystal formation. Kidney tubules crystals are almost always formed by Ig light chain of κ subtype due to resistance of their variable domain to proteolysis by cathepsin B, a lysosomal protease present in proximal tubule cells [6], in contrast to λ subtype light chains which are less prone to crystallization. IgD myeloma has a unique light chain λ variable domain somatic hypermutation [7] conferring resistance to proteolysis and new interaction sites favoring crystal formation. The rapidly worsening renal function and oliguria in our patient suggests tubular injury that might be caused by light chain λ crystallization (not confirmed by renal biopsy). The bias for λ light chain expression with a reversed light chain ratio is a characteristic feature of IgD MM. Shimamoto et al reported it in 82% of patients with Ig D myeloma [8].

Over the last decade, multiple randomized trials have shown the superiority of novel immunomodulatory agents (thalidomide) and proteasome inhibitors (bortezomib) in combination with ASCT have shown over standard therapy (melphalan, vincristine, adriamycin, and dexamethasone) [9].

Patients often remain on dialysis despite better control of the hematological component of myeloma. Extracorporeal removal of FLCs with plasmapheresis is theoretically a treatment option by reducing the level of free light chain and consequently reducing its nephrotoxicity. However, its impact on patient prognosis and survival remains to be demonstrated [10].

### Conclusion

IgD MM is rare (1.5-2%), has onset at a younger age with advanced disease at time of diagnosis and poor prognosis compared with other types of MM. All MM patients with light chain proteinuria and small or absent M-spike should be evaluated for IgD MM isotype. More trials with HDT/ASCT are needed to formulate therapeutic guideline for better outcome.

## References

[1] Blade J, Kyle RA (1999). Nonsecretory myeloma, immunoglobulin D myeloma, and plasma cell leukemia. Hematol Oncol Clin North Am.

[2] Dana AP, Ahmar CA, Clapp WL, Ross EA (2011). A new form of myeloma "kidney": shortened hemofilter survival and implications for membrane filtration plasmapheresis. Clin Nephrol.

[3] Blade J, Lust JA, Kyle RA (1994). Immunoglobulin D multiple myeloma: presenting features, response to therapy, and survival in a series of 53 cases. J Clin Oncol.

[4] Maisnar V, Hajek R, Scudla V, Gregora E, Buchler T, Tichy M, Kotoucek P (2008). High-dose chemotherapy followed by autologous stem cell transplantation changes prognosis of IgD multiple myeloma. Bone Marrow Transplant.

[5] Kuliszkiewicz-Janus M, Zimny A, Sokolska V, Sasiadek M, Kuliczkowski K (2005). Immunoglobulin D myeloma--problems with diagnosing and staging (own experience and literature review). Leuk Lymphoma.

[6] Messiaen T, Deret S, Mougenot B, Bridoux F, Dequiedt P, Dion JJ, Makdassi R (2000). Adult Fanconi syndrome secondary to light chain gammopathy. Clinicopathologic heterogeneity and unusual features in 11 patients. Medicine (Baltimore).

[7] Arpin C, de Bouteiller O, Razanajaona D, Fugier-Vivier I, Briere F, Banchereau J, Lebecque S (1998). The normal counterpart of IgD myeloma cells in germinal center displays extensively mutated IgVH gene, Cmu-Cdelta switch, and lambda light chain expression. J Exp Med.

[8] Shimamoto Y, Anami Y, Yamaguchi M (1991). A new risk grouping for IgD myeloma based on analysis of 165 Japanese patients. Eur J Haematol.

[9] Sekiguchi N, Takezako N, Nagata A, Wagatsuma M, Noto S, Yamada K, Miwa A (2011). Successful treatment of immunoglobulin D myeloma by bortezomib and dexamethasone therapy. Intern Med.

[10] Madore F (2015). Plasmapheresis in cast nephropathy: yes or no?. Curr Opin Nephrol Hypertens.

